# LC-MS/MS-based metabolic profiling: unraveling the impact of varying degrees of curing on metabolite transformations in tobacco

**DOI:** 10.3389/fpls.2024.1473527

**Published:** 2024-11-12

**Authors:** Kesu Wei, Xuling Chen, Zhijun Cheng, Heng Wang, Feng Wang, Lei Yang, Shengjiang Wu, Yijun Yang, Yonggao Tu, Yan Wang, Chenggang Liang

**Affiliations:** ^1^ Upland Flue-cured Tobacco Quality and Ecology Key Laboratory, China National Tobacco Corporation (CNTC), Guizhou Academy of Tobacco Science, Guiyang, China; ^2^ College of Life Science, Guizhou Normal University, Guiyang, China; ^3^ China Tobacco Hunan Industrial Co. Ltd, Changsha, China; ^4^ School of Biological and Environmental Engineering, Guiyang College, Guiyang, China

**Keywords:** tobacco, curing degree, curing stage, metabolite transformation, flavonoids, sugar

## Abstract

The curing process regulates metabolite transformations of leaves and significantly influences the formation of tobacco quality. This study investigated the major physicochemical compositions and metabolic profiles under normal curing (NC), excessive curing (EC), and insufficient curing (IC) treatments. The results indicated that the contents of nicotine, nitrogen, potassium, and chlorine remained stable among treatments, while the sugar content in EC was significantly lower than in IC. LC-MS/MS identified 845 metabolites, with flavonoids as the most abundant class. Comparative analyses identified a series of differentially expressed metabolites (DEMs) among fresh leaf, NC, EC, and IC leaves at the end of 42°C, 54°C, and 68°C, respectively. At the end of 68°C, 256 up-regulated and 241 down-regulated common DEMs across treatments were isolated in comparison to fresh leaf, underscoring the consistency of metabolic changes during curing. Notably, nonivamide varied markedly across treatments, suggesting its potential as a key curing indicator. NC_68°C displayed 11 up-regulated and 17 down-regulated unique DEMs, differing from EC_68°C and IC_68°C, suggesting their potential availability in evaluating tobacco leaf quality. KEGG pathway analysis revealed temporal shifts in metabolic pathways, particularly those involved in secondary metabolite biosynthesis (such as flavonoids, flavones, flavonols) and amino acid metabolism, during the transition from yellowing to color-fixing. Correlation analysis isolated the top 25 DEMs correlated with curing degree and stage, which might play pivotal roles in the curing process and could serve as potential biomarkers for assessing curing degree and stage. Specifically, D-(+)-cellobiose displayed the strongest negative correlation with curing degree, while 5,7-dihydroxychromone exhibited the highest positive correlation coefficient. Furthermore, curcurbitacin IIa showed the highest positive correlation with curing stage, followed by hesperetin and 8-shogaol. Additionally, random forest analysis emphasized morellic acid as a core molecular metabolite across curing degrees, suggesting its potential as a biomarker. Debiased sparse partial correlation (DSPC) network analysis further pinpointed hispidulin as a key metabolite, underscoring its significance in elucidating flavonoid metabolism during the curing process. Collectively, this study enhances the understanding of metabolite transformations underlying tobacco curing processes and provides a valuable reference for optimizing curing strategies to achieve desired outcomes.

## 1 Introduction

Tobacco (*Nicotiana tabacum* L.), a globally economically significant crop, not only plays a pivotal role in agricultural economies but also holds a unique position in aromatic industrial applications (Mavroeidis et al., 2024). Tobacco leaves undergo a series of intricate processing steps, with curing being a critical phase that significantly impacts the final quality and flavor of the tobacco (Li et al., 2022). Curing involves carefully controlled conditions of temperature, humidity, and airflow, aimed at gradually drying the leaves while promoting desired chemical reactions to enhance the aroma, reduce moisture content, and prepare the leaves for further processing and consumption (Bacon et al., 1952). Notably, the flue-curing process, which typically lasts 6-7 days, involves the application of artificial heat to elicit a cascade of biochemical reactions. These reactions transform the leaf color and chemical composition, ultimately dictating the quality and marketability of the final product (Abubakar et al., 2000; Chen et al., 2019; Meng et al., 2024).

The curing process is typically characterized by three pivotal stages: the yellowing stage, the color fixing stage, and the dry tendon stage (Zou et al., 2019; Meng et al., 2024). Specifically, the degree of yellowing during this process is a critical parameter that not only influences leaf visual appeal but also plays a pivotal role in determining the chemical profile and subsequent industrial applications (Zou et al., 2019). Therefore, the formation and accumulation of aromatic compounds, polyphenols, and other flavor-related chemicals during this process have been extensively studied, as they contribute significantly to the smoking characteristics and overall quality of the cured tobacco (Vaughan et al., 2008; Lim et al., 2022; Zou et al., 2023). It is widely acknowledged that the color of flue-cured tobacco, serving as a crucial quality indicator, exhibits a strong correlation with natural pigment content during the curing process (Meng et al., 2024). Leaf performance is commonly utilized to evaluate and guide decisions related to tobacco quality and industrial applicability (Xin et al., 2023).

The curing process of tobacco involves various aspects and factors, including drying methods, temperature, humidity, duration, draft fan control, and the use of exogenous additives. Studies on this process have elucidated the transformation law of major chemical compositions in different types of tobacco under various flue-curing methods, indicating the feasibility of combining multiple curing methods to enrich style characteristics and potentially enhance tobacco quality (Bacon et al., 1952). In comparison to oven-drying, flue-curing leads to significant decreases in four plastid pigments (lutein, chlorophyll A, chlorophyll B, β-carotene) and notable increases in six polyphenol substances (neochlorogenic acid, chlorogenic acid, caffeic acid, chrysatropic acid, rutin, kaempferol) (Zong et al., 2022). Optimization of wind speed parameters through the use of a heat pump-powered curing barn and a three-stage curing process has been shown to significantly enhance the baking quality of tobacco leaves, promoting the accumulation of aroma substances and the degradation of macromolecular substances (Sun et al., 2023). Furthermore, the pectinase preparation derived from *Bacillus amyloliquefaciens* W6-2 has been found to effectively enhance the quality of flue-cured tobacco, as evidenced by improvements in aroma, sweetness, and smoothness, along with alterations in the levels of macromolecules and volatile components (Weng et al., 2024). The application of starch-degrading bacteria has been shown to significantly improve tobacco leaf quality by reducing macromolecule content, increasing water-soluble total sugar and reducing sugar levels, and enhancing the production of crucial volatile aroma components (Gong et al., 2023). Another study revealed that the application of γ-PGA in flue-cured tobacco leaves influenced the accumulation and transformation of carbon and nitrogen compounds, regulating the carbon and nitrogen metabolic processes during leaf growth and ultimately affecting tobacco leaf quality (Gao et al., 2023). Additionally, the presence of stems has been found to alter leaf metabolism, prevent browning, and enhance starch degradation during flue-curing, ultimately resulting in lower starch content in leaves (Meng et al., 2022).

Recently, metabolomics has emerged as a powerful tool for comprehensively profiling the metabolic shifts that occur during various biological processes. For instance, GC-MS analysis has identified 128 flavor chemicals in tobacco flavor capsules, with menthol as the dominant component and the carcinogenic compound pulegone also detected, although exposure margins were below safety thresholds (Lim et al., 2022). LC-MS/MS analysis identified 259 and 178 differentially expressed metabolites (DEMs) between 0.4 M sucrose-treated and control tobacco leaves in the early and late stages of air-curing, respectively, revealing alterations in carbohydrate and amino acid metabolism that promote normal chlorophyll degradation and mitigate the green spot phenomenon through sucrose treatment (Li et al., 2023). Furthermore, GC-MS and CE-MS analyses have uncovered significant metabolic differences between growing districts, with a complex carbon and nitrogen metabolic network modulated by environmental factors (Zhao et al., 2015). A recent study elucidated the key chemical components contributing to the honey-sweet and burnt aroma characteristics of Shandong flue-cured tobacco, identifying 29 aroma precursors positively correlated with sensory quality, thereby providing guidance for targeted improvement and precise regulation of flavor-style characteristics (Li et al., 2024). UPLC-Q-TOF MS and GC-MS identified alpha-cembratriene-diol, beta-cembratriene-diol, sucrose esters III, and cembratriene-diol oxide as primary contributors to antifungal activity, providing insights for the development of botanical pesticides and multipurpose utilization of tobacco germplasms (Liu et al., 2024).

It is widely recognized that curing technology significantly influences the transformation of leaf metabolites, which in turn directly affects tobacco quality and price. Yunyan 87, a highly regarded tobacco cultivar, is notable for its wide adaptability, robust stress resistance, stable yield, ease of curing, and superior quality, which is characterized by a striking golden to orange-yellow color, rich oil content, high gloss, and moderate thickness, all collectively contributing to its prominent market value (Li et al., 2001). However, the metabolite transformation across different degrees of curing remains incompletely understood. Typically, the assessment of whether curing meets the standard relies heavily on visual data, lacking quality indicators such as metabolites. Therefore, the primary objective of this study is to conduct a detailed analysis of metabolite profiles at three typical stages of the flue-curing process in Yunyan 87 using the LC-MS/MS platform, specifically comparing normal curing (NC), excessive curing (EC), and insufficient curing (IC) treatments, and to investigate the differential transformation of key metabolites, which is expected to provide a comprehensive understanding of how varying degrees of curing can influence the transformation of metabolites in tobacco and offer valuable insights for optimizing curing practices.

## 2 Results and discussion

### 2.1 Physicochemical composition

To identify the differences in major physicochemical compositions among various treatments, tobacco leaf samples were collected and analyzed following the completion of the 68°C curing process (**Table 1**). Despite the absence of statistical differences in leaf weight and leaf density across treatments, EC demonstrated slightly lower levels of both parameters compared to NC and IC. Nitrogen content in tobacco leaves exerts multifaceted effects on quality, primarily as a constituent of the nicotine molecule, which directly influences nicotine concentrations and, consequently, the overall quality of flue-cured tobacco leaves (Henry et al., 2019). As shown in **Table 1**, both nitrogen and nicotine content in tobacco were largely unaffected by the different treatments, suggesting minimal alterations in the conversion of nitrogen and nicotine, despite variations in curing temperature and duration. Potassium and chlorine contents also play crucial roles in determining the quality of tobacco leaves (Attoe, 1946). The potassium and chlorine contents are found to be relatively stable in air-cured, flue-cured, and sun-cured tobacco leaves (Chen et al., 2021). Similarly, these components did not exhibit significant changes in response to the treatments. It is well established that sugars in tobacco leaves play a significant role in determining quality, as they influence the formation of harmful compounds and smoking properties. High temperatures in flue and sun curing elevate final sugar content, whereas low temperatures in air curing decrease sugar levels (Banožić et al., 2020). A notable observation was the variation in sugar content among the treatments, with EC exhibiting the lowest sugar content, followed by NC and IC. The sugar content of EC was significantly lower than that of IC. Therefore, variations in sugar transformation due to different curing conditions might have implications for the final quality of tobacco.

**Table 1:** The major physicochemical compositions of tobacco under different treatments.

Treatment	Leaf weight (g)	Leaf density (g/m^2^)	Sugar content (%)	Nicotine content (%)	Nitrogen content (%)	Potassium content (%)	Chlorine content (%)
NC	8.5 ± 0.7a	75.7 ± 1.5a	19.0 ± 2.8ab	3.51 ± 0.08a	2.44 ± 0.17a	1.47 ± 0.02a	0.25 ± 0.03a
EC	7.8 ± 0.5a	68.7 ± 5.0a	15.9 ± 3.3b	3.42 ± 0.32a	2.33 ± 0.10a	1.52 ± 0.07a	0.27 ± 0.04a
IC	8.8 ± 0.4a	74.0 ± 6.1a	21.4 ± 1.9a	3.46 ± 0.12a	2.40 ± 0.09a	1.58 ± 0.10a	0.25 ± 0.01a

NC, normal curing; EC, excessive curing; IC, insufficient curing. Different lowercase letters indicate significant differences among treatments according to One-way ANOVA.

### 2.2 Multivariate analysis of metabolomics

**Figure 1:**
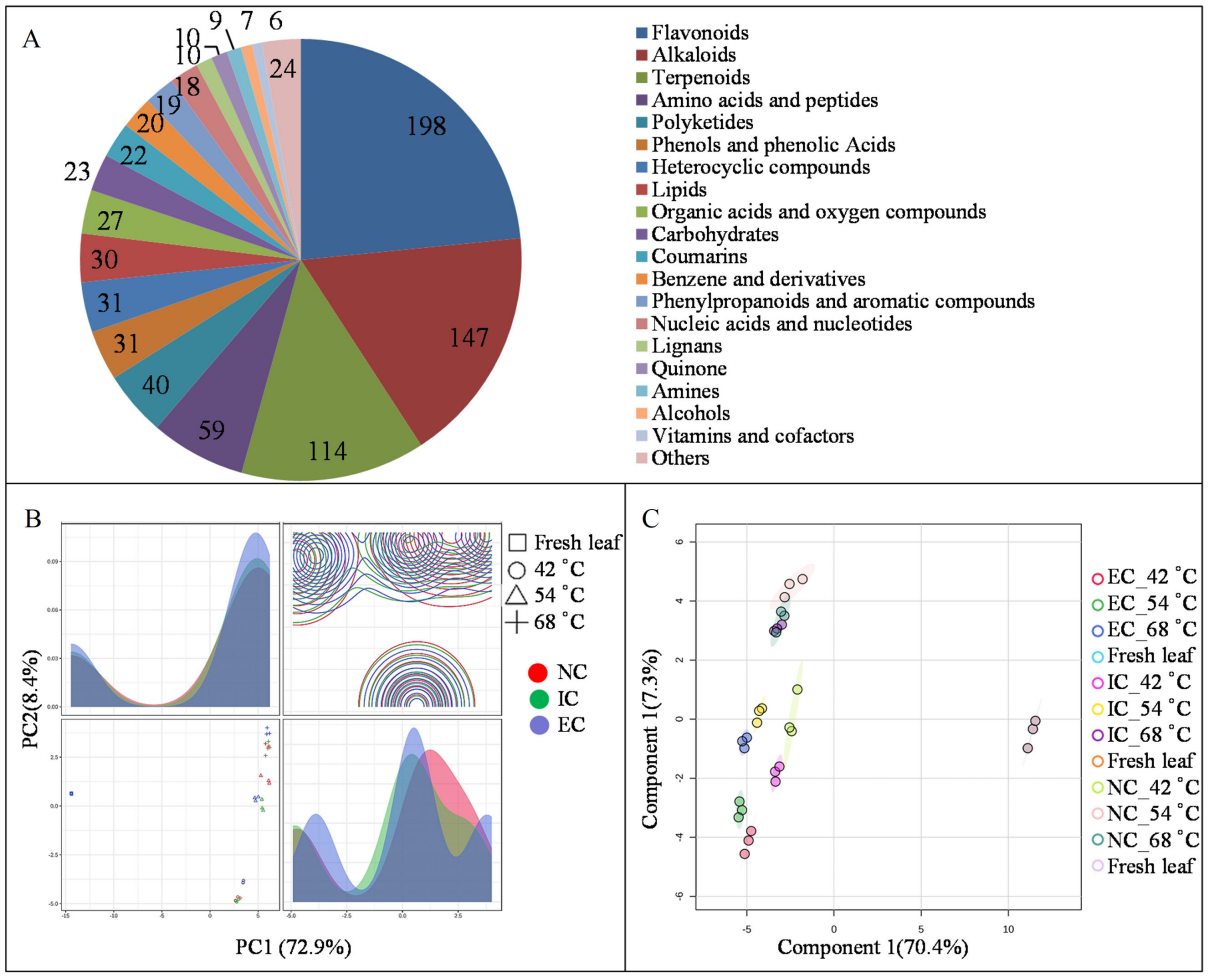
The groups of metabolite **(A)**, PCA score **(B)**, and OPLS-DA score **(C)** by LC-MS/MS analysis. EC represents excessive curing, IC represents insufficient curing, NC represents normal curing, the same as below.

Metabolomics is a powerful tool for uncovering the rules of metabolite transformation during tobacco leaf curing (Zong et al., 2022). To gain insights into the impact of different curing degrees on metabolome dynamics in tobacco leaves, metabolomics analysis was conducted using an LC–MS/MS platform. A total of 845 metabolites were identified and predominantly categorized into 20 distinct groups, including 198 flavonoids, 147 alkaloids, 114 terpenoids, 59 amino acids and peptides, 31 phenols and phenolic acids, 31 polyketides, 30 lipids, 27 organic acids and oxygen compounds, 23 carbohydrates, 22 coumarins, 20 benzene derivatives, 19 phenylpropanoids, and aromatic compounds (**Figure 1A**). Principal Component Analysis (PCA) revealed distinct metabolome profiles between the NC, IC, and EC treatments at 42, 54, and 68°C, with PC1 and PC2 explaining 72.9% and 8.4% of the variance, respectively (**Figure 1B**). Subsequently, OPLS-DA analysis was employed to enhance the differentiation among the NC, IC, and EC treatments, confirming the suitability of the model for identifying differentially expressed metabolites (DEMs), with Component 1 and Component 2 accounting for 70.4% and 7.3% of the variance, respectively (**Figure 1C**).

### 2.3 Analysis of DEMs

**Figure 2:**
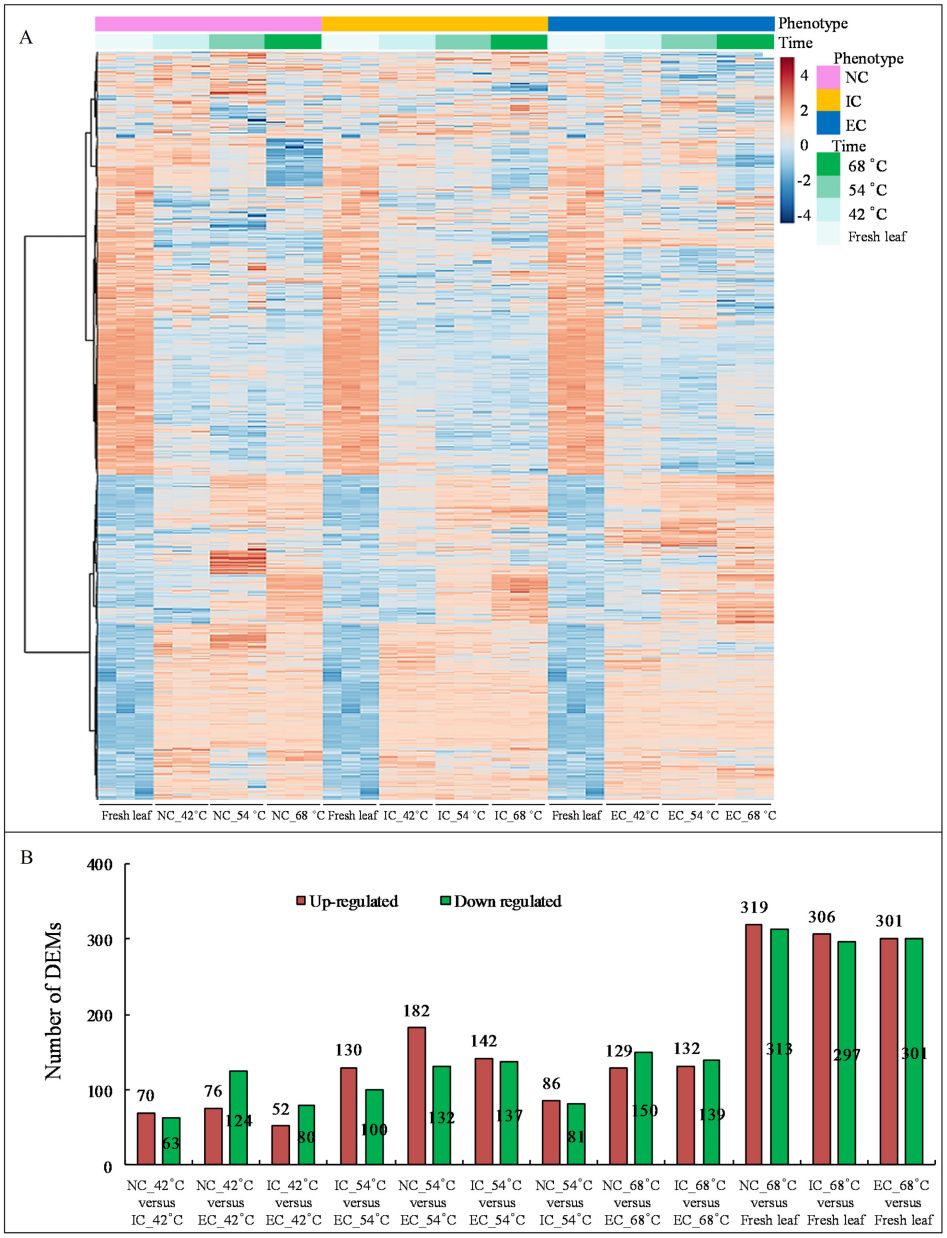
The heatmap of metabolites **(A)** and the number of differentially expressed merabolites by pairwise comparison analyses **(B)**.

**Figure 3:**
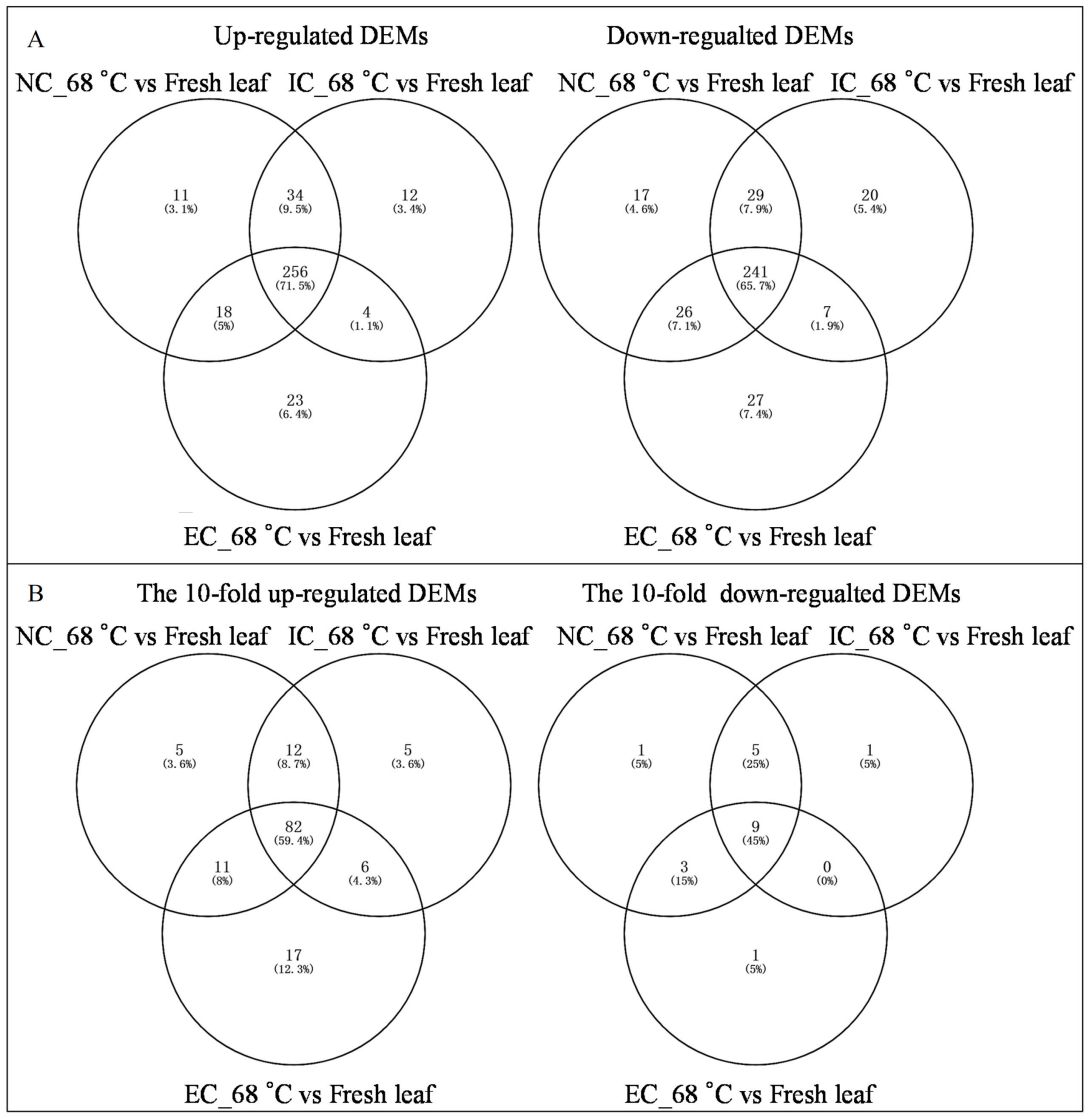
Venn diagram of differential expressed metabolites **(A)** and the 10-fold differential expressed metabolites **(B)** between tobacco cured leaves subjected to different curing treatments and fresh leaf.

The heat map demonstrated significant variations in metabolite levels during curing (**Figure 2A**). Pairwise comparison analyses revealed 319 up-regulated and 313 down-regulated DEMs (*p*-value ≤ 0.05 and VIP value ≥ 1) in the comparison of NC_68°C vs fresh leaf, 306 up-regulated and 297 down-regulated DEMs in IC_68°C vs fresh leaf, and 301 up-regulated and 301 down-regulated DEMs in EC_68°C vs fresh leaf (**Figure 2B**). Among these, 256 up-regulated and 241 down-regulated common DEMs were consistently observed across NC_68°C vs fresh leaf, IC_68°C vs fresh leaf, and EC_68°C vs fresh leaf (**Figure 3A**). Notably, 91 DEMs exhibited at least a 10-fold change (82 up-regulated and 9 down-regulated), indicating consistent and significant changes during the curing process (**Figure 3B**; **Supplementary Table S1**).

The top 20 fold-change DEMs during curing under NC condition are present in **Table 2**. At the yellowing stage, notable increases exceeding 100-fold were detected in isochlorogenic acid B, 3,5-dicaffeoylquinic acid, 1-caffeoylquinic acid, isochlorogenic acid C, and toosendanin. Conversely, silychristin, N-acetyl-L-carnosine, isoanhydroicaritin, nonivamide, fluoxetine, and adenine exhibited decreases of more than 100-fold. During color fixing, scopolamine hydrobromide, scopolamine HBr trihydrate, brevifolincarboxylic acid, and 3-nitro-L-tyrosine increased greater than 50-fold, while acesulfame, isoschaftoside, vicenin III, and ipecoside underwent comparable declines. At the dry tendon stage, polydatin levels surged by over 50-fold, but decursinol, Robinin, apigenin 7-o-(2g-rhamnosyl) gentiobioside, scopolamine hydrobromide, luteolin 3’,7-di-o-glucoside, scopolamine HBr trihydrate, 3-nitro-L-tyrosine, and D-(-)-penicillamine decreased by more than 100-fold (**Table 2**). Furthermore, 6 DEMs demonstrated substantial changes across two stages, with 4 of them (scopolamine hydrobromide, scopolamine HBr trihydrate, 3-nitro-L-tyrosine, and D-(-)-penicillamine) experiencing a sharp rise in the color fixing stage, followed by a steep decline in the dry tendon stage.

**Table 2:** The top 20 fold-change DEMs at three pivotal stages under normal curing condition.

Yellowing stage (NC 42°C vs Fresh leaf)		Color fixing stage (NC 54°C vs NC 42°C)		Dry tendon stage (NC 68°C vs NC 54°C)	
Metabolite	Fold	Metabolite	Fold	Metabolite	Fold
Isochlorogenic acid B	318.95	**Scopolamine hydrobromide**	130.09	Polydatin	50.17
3,5-dicaffeoylquinic acid	279.94	**Scopolamine HBr trihydrate**	112.86	Byakangelicol	9.87
1-caffeoylquinic acid	264.48	Brevifolincarboxylic acid	89.9	Vicine	8.75
Isochlorogenic acid C	234.44	**3-nitro-L-tyrosine**	70.44	(S)-3-(allylsulphinyl)-L-alanine	7.22
Toosendanin	103.54	Ethylenediaminetetraacetic acid	34.97	Deoxyandrographolide	7.01
5-iodo-2’-deoxyuridine	94.33	Paeoniflorin	31.95	**Acesulfame**	5.49
Periplocymarin	89.38	Monocrotaline	31.64	Piperonyl acetone	5.26
7-hydroxycoumarin	88.56	Valsartan	22.18	Glycitin	5.20
Gentiopicrin	86.91	**D-(-)-penicillamine**	21.00	Lithosprmoside	5.06
Apigenin-7-o-beta-d-glucoside	78.48	Leonurine	17.47	Amarogentin	4.99
Bisdemethoxycurcumin	0.11	Ranaconitine	0.31	Marmesin	0.11
L-isoleucine	0.11	Daidzin	0.28	Polyphyllin VI	0.11
Bilobalide	0.11	Tyramine	0.28	Decursinol	0.09
p-Hydroxy-cinnamic acid	0.11	Deoxyadenosine monophosphate	0.22	Kaempferol-3-o-robinoside-7-o-rhamnoside (Robinin)	0.07
Silychristin	0.09	**Fluoxetine**	0.21	Apigenin 7-o-(2G-rhamnosyl) gentiobioside	0.07
N-acetyl-L-carnosine	0.08	Acetylcorynoline	0.21	**Scopolamine hydrobromide**	0.07
Isoanhydroicaritin	0.07	**Acesulfame**	0.17	Luteolin 3’,7-di-o-glucoside	0.07
Nonivamide	0.07	Isoschaftoside	0.09	**Scopolamine HBr trihydrate**	0.06
**Fluoxetine**	0.07	Vicenin III	0.06	**3-Nitro-L-tyrosine**	0.04
Adenine	0.06	Ipecoside	0.05	**D-(-)-Penicillamine**	0.03

The bolded fonts represent the common DEMs among treatments.

In contrast, each pairwise comparison identified unique DEMs, indicative of distinct metabolic responses to the various treatments. Specifically, NC_68°C vs fresh leaf showed 11 up-regulated and 17 down-regulated DEMs, IC_68°C vs fresh leaf had 12 up-regulated and 20 down-regulated, while EC_68°C vs fresh leaf exhibited 23 up-regulated and 27 down-regulated DEMs (**Figure 3B**; **Supplementary Table S1**). Further pairwise analyses uncovered shared and exclusive DEMs between treatment comparisons. For instance, 34 up-regulated and 26 down-regulated DEMs were common to NC_68°C vs fresh leaf and IC_68°C vs fresh leaf, while 18 up-regulated and 29 down-regulated DEMs were shared between NC_68°C vs fresh leaf and EC_68°C vs fresh leaf. The comparison between IC_68°C vs fresh leaf and EC_68°C vs fresh leaf showed 4 up-regulated and 7 down-regulated DEMs (**Figure 3A**). Importantly, unique sets of metabolites with more than 10-fold differential expression were identified, with corrections made to avoid repetition: 5, 5, and 17 up-regulated DEMs (each unique to a comparison), along with 1, 1, and 0 down-regulated DEMs, respectively (**Figure 3B**; **Supplementary Table S1**). Additionally, in examining the intersections among the pairwise groups, distinct patterns emerged, with 12, 11, and 6 up-regulated DEMs, and 3, 5, and 0 down-regulated common DEMs observed, respectively, providing insights into the overlap and divergence of metabolic responses under different curing degrees.

During curing, metabolite levels exhibited moderate variation across treatments at identical temperatures. Comparative analyses at 42, 54, and 68°C revealed the following differentially expressed metabolites (DEMs): 70, 130, and 86 up-regulated, and 63, 100, and 81 down-regulated DEMs in NC vs IC comparison; 76, 182, and 129 up-regulated, and 124, 137, and 150 down-regulated DEMs in NC vs EC comparison; and 52, 142, and 132 up-regulated, with 80, 137, and 139 down-regulated DEMs in IC vs EC comparison, respectively (**Figure 2B**).

Within the NC vs IC group, 5 (2 up-regulated and 3 down-regulated), 39 (25 up-regulated and 14 down-regulated), and 3 (2 up-regulated and 1 down-regulated) DEMs changed at least 3-fold at 42, 54, and 68°C, respectively. In the NC vs EC group, 23 (2 up-regulated and 21 down-regulated), 65 (41 up-regulated and 24 down-regulated), and 21 (5 up-regulated and 16 down-regulated) DEMs changed at least 3-fold at 42, 54, and 68°C, respectively. And in the IC vs EC group, 23 (2 up-regulated and 8 down-regulated), 22 (9 up-regulated and 13 down-regulated), and 31 (9 up-regulated and 22 down-regulated) DEMs changed at least 3-fold at 42, 54, and 68°C, respectively (**Supplementary Table S2**).

The top 20 fold-change DEMs at the end of curing among treatments are presented in **Table 3**. In the comparison of NC_68°C vs. IC_68°C, 7-ethyl-10-hydroxy-camptothecin (3.89-fold), prim-o-glucosylcimifugin (3.08-fold), and cimetidine (2.88-fold) were the most up-regulated DEMs, while di-o-methylquercetin (0.26-fold) was the most down-regulated. When comparing IC_68°C to EC_68°C, S-(-)-carbidopa (30.46-fold), acyclovir (8.84-fold), and acetylcorynoline (5.34-fold) showed the highest up-regulation, whereas several DEMs, including nonivamide (0.10-fold), myricetin (0.12-fold), liquiritin (0.13-fold), and valsartan (0.16-fold), were significantly down-regulated. In the NC_68°C vs. EC_68°C comparison, S-(-)-carbidopa (7.92-fold), cimetidine (6.88-fold), and acyclovir (5.23-fold) were the most up-regulated DEMs, while octyl gallate (0.14-fold), liquiritin (0.17-fold), myricetin (0.17-fold), and valsartan (0.18-fold) were the most down-regulated (**Table 3**). Furthermore, 15 common DEMs were detected among treatments (**Table 3**). Notably, nonivamide exhibited substantial variations across the three treatments, with the highest levels observed in EC_68°C, moderate levels in NC_68°C, and the lowest levels in IC_68°C, suggesting that nonivamide might be served as a key metabolite for assessing the degree of curing.

**Table 3:** The top 20 fold-change DEMs at the end of curing among treatments.

NC_68°C vs IC_68°C		IC_68°C vs EC_68°C		NC_68°C vs EC_68°C	
Metabolite	Fold	Metabolite	Fold	Metabolite	Fold
**7-ethyl-10-hydroxy-camptothecin**	3.89	**S-(-)-carbidopa**	30.46	**S-(-)-Carbidopa**	7.92
Prim-o-glucosylcimifugin	3.08	**Acyclovir**	8.84	**Cimetidine**	6.88
**Cimetidine**	2.88	**Acetylcorynoline**	5.34	**Acyclovir**	5.23
Glabridin	2.83	Picfeltarraenin IB	4.63	Ipecoside	3.71
Laetanine	2.8	**Byakangelicol**	3.84	**Glycitin**	3.48
Bavachin	2.75	**Deoxyandrographolide**	3.69	Adenosine cyclophosphate	2.98
Isobavachalcone	2.73	14-deoxyandrographolide	3.67	Isoschaftoside	2.95
Homoplantaginin	2.70	Gentiopicrin	3.23	**Deoxyandrographolide**	2.91
Chrysoeriol 5-o-hexoside	2.70	**Glycitin**	3.11	6’’-O-Acetylglycitin	2.88
Emodin-3-methyl ether/Physcion	2.47	Apigenin-7-o-beta-d-glucoside	0.21	**Byakangelicol**	2.88
Diosmetin-7-o-beta-d-glucopyranoside	2.46	Genistin	0.21	**10-Hydroxydecanoic acid**	0.25
Tectoridin	2.43	**Chrysosplenetin B**	0.21	**Casticin**	0.24
**Nonivamide**	2.43	**Casticin**	0.18	**Chrysosplenetin B**	0.23
Morellic acid	0.47	**Valsartan**	0.16	**Nonivamide**	0.23
Tetrahydropiperine	0.44	**7-ethyl-10-hydroxy-camptothecin**	0.15	Atropine sulfate monohydrate	0.22
**Acetylcorynoline**	0.43	**Octyl gallate**	0.15	Mangiferin	0.21
L-alanyl-L-phenylalanine	0.42	**Liquiritin**	0.13	**Valsartan**	0.18
Toosendanin	0.41	**Myricetin**	0.12	**Liquiritin**	0.17
**S-(-)-carbidopa**	0.41	**10-Hydroxydecanoic acid**	0.11	**Myricetin**	0.17
Di-o-methylquercetin	0.26	**Nonivamide**	0.10	**Octyl gallate**	0.14

The bolded fonts represent the common DEMs among treatments.

### 2.4 Analysis of correlation

**Figure 4:**
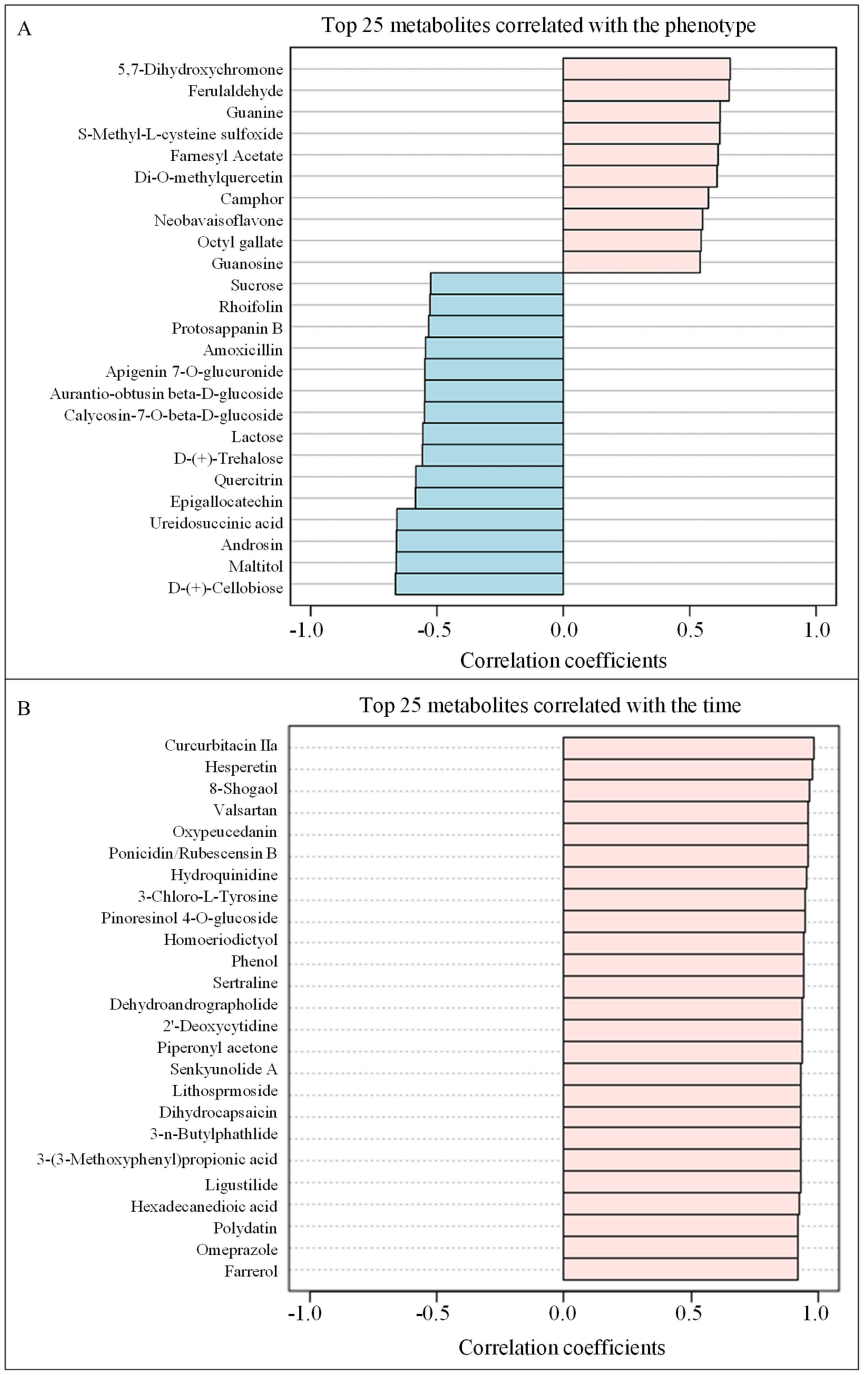
The correlation of the top 25 metabolites with the phenotype **(A)** and the time **(B)**.

The top 25 metabolites correlated with the phenotype (curing degree) were isolated, with 10 positively correlated and 15 negatively correlated (**Figure 4A**). Flavonoids, the most abundant class of metabolites in tobacco leaves, possess diverse biological functions (Hu et al., 2021). The contents of flavonoid metabolites exhibited a significant decrease throughout the air-curing period (Geng et al., 2024). Notably, 5 flavonoids and 4 carbohydrates were negatively correlated, whereas 3 flavonoids showed positive correlation with the phenotype (**Figure 4A**). D-(+)-Cellobiose displayed the strongest negative correlation, while 5,7-Dihydroxychromone exhibited the highest positive correlation coefficient. Carbohydrates serve as vital precursors for aroma and bioactive compounds (Banožić et al., 2020). The observed negative correlation between sucrose content and phenotype, consistent with the sugar content data in **Table 1**, underscores the profound influence of curing degree on tobacco leaf carbohydrate metabolism.

Furthermore, the top 25 metabolites were positively correlated with the time (curing stage), encompassing a diverse array of compounds crucial to tobacco quality (**Figure 4B**). It is well established that terpenoids (Demole and Enggist, 1975), phenols and phenolic acids (Zou et al., 2021), heterocyclic compounds (Higashio and Shoji, 2004), amino acids and peptides (Wang et al., 2020), and alkaloids (Shoji et al., 2024) are well-known contributors to the aroma, flavor, and overall sensory quality of tobacco leaves. Notably, curcurbitacin IIa showed the highest positive correlation with time, followed closely by hesperetin and 8-shogaol, indicating their potential significance in the curing process.

### 2.5 Analysis of Kyoto encyclopedia of genes and genomes pathways

**Figure 5:**
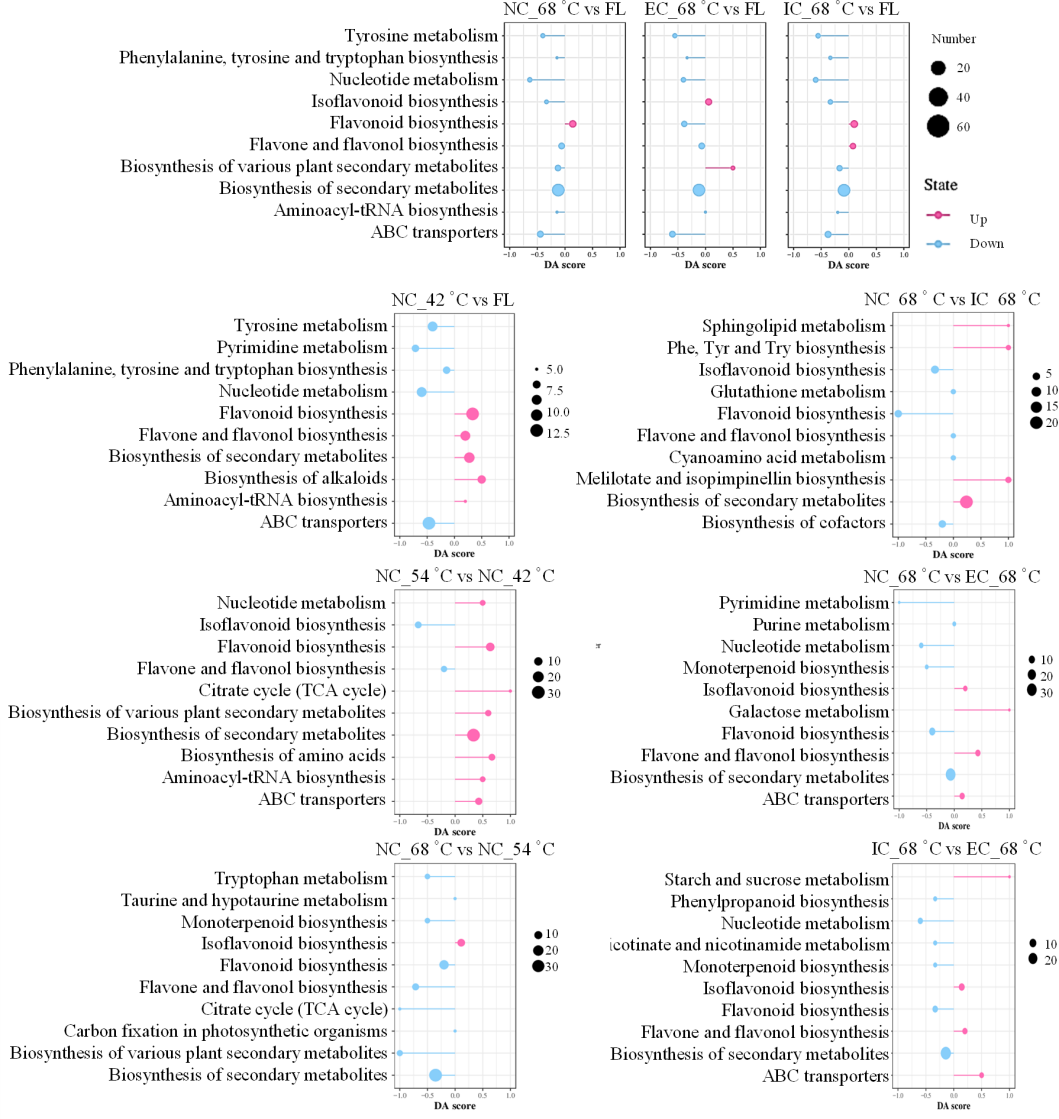
Analysis of differential expressed metabolites using KEGG pathway analysis. FL represents fresh leaf.

The KEGG analysis revealed a striking similarity in metabolic strategies employed under varying curing degrees, as evidenced by the collective enrichment of the same top 10 metabolic pathways among the DEMs derived from the comparisons of NC_68°C vs fresh leaf, EC_68°C vs fresh leaf, and IC_68°C vs fresh leaf (**Figure 5**). Consistent with previous findings (Yang et al., 2023), this study also observed a prominent enrichment of DEMs in the flavonoid biosynthesis pathway and amino acid metabolism. Specifically, the synthesis of various flavonoid metabolites, such as isoflavonoids, flavonoids, flavones, and flavonols, along with amino acid metabolism, were enriched. However, despite these shared enrichments, closer inspection revealed distinct patterns within the flavonoid biosynthesis pathways. Notably, the isoflavonoid biosynthesis pathway, flavone and flavonol biosynthesis pathway exhibited variations among different treatments, indicating that the flavonoid metabolism was indeed influenced by the specific curing degrees.

The top 10 metabolic pathways covered by the DEMs in the comparisons of NC_42°C vs fresh leaf, NC_54°C vs NC_42°C, and NC_68°C vs NC_54°C reflected the distinct treatment effects. The yellowing stage represents the apex of primary chemical component conversion, during which numerous metabolites within tobacco leaves undergo profound alterations (Zou et al., 2019). A comparative analysis between NC_42°C and fresh leaf, revealed a notable enrichment of up-regulated metabolic pathways pertaining to secondary metabolite biosynthesis, particularly flavonoid, flavone, and flavonol biosynthesis, as well as alkaloid biosynthesis. Conversely, pathways such as ABC transporters, tyrosine metabolism, and nucleotide metabolism were down-regulated, indicating a shift towards enhanced secondary metabolite synthesis accompanied by augmented amino acid degradation. Further investigation comparing NC_54°C to NC_42°C showed a distinct pattern, with only two pathways (isoflavonoid biosynthesis and flavone/flavonol biosynthesis) being down-regulated, while the remaining eight pathways, including those related to secondary metabolite and flavonoid biosynthesis, were up-regulated. This suggested a continuation and intensification of metabolic activity in these pathways during the transition from yellowing to color fixing. However, in the comparison between NC_68°C and NC_54°C, while isoflavonoid biosynthesis was up-regulated, pathways associated with secondary metabolite and flavonoid biosynthesis, as well as tryptophan metabolism, exhibited down-regulation. These findings implied that metabolic pathways, especially those linked to secondary metabolites and flavonoids, undergo a surge during the yellowing to color fixing transition but might experience a subsequent decline as leaves progress towards the dry tendon stage. This dynamic regulation of metabolic pathways highlighted the intricacies and temporal specificity involved in tobacco curing processes.

Additionally, the results of the comparisons between different treatments at 68°C revealed intricate regulatory patterns in metabolic pathways. Specifically, when comparing NC_68°C to IC_68°C, DEMs were enriched in up-regulated biosynthesis of secondary metabolites and down-regulated isoflavonoid biosynthesis. In contrast, NC_68°C vs EC_68°C showed DEMs enriched in up-regulated flavone and flavonol biosynthesis and down-regulated biosynthesis of secondary metabolites. Furthermore, IC_68°C vs EC_68°C showed DEMs enriched in up-regulated isoflavonoid biosynthesis and down-regulated biosynthesis of secondary metabolites. These findings suggested that the curing degrees significantly impacted the metabolic landscape of tobacco leaves, particularly with respect to secondary metabolite biosynthesis. The differential regulation of specific pathways, such as isoflavonoid, flavone, and flavonol biosynthesis, highlighted the intricate interplay between treatments and metabolic fluxes during tobacco curing.

### 2.6 Analysis of random forest

**Figure 6:**
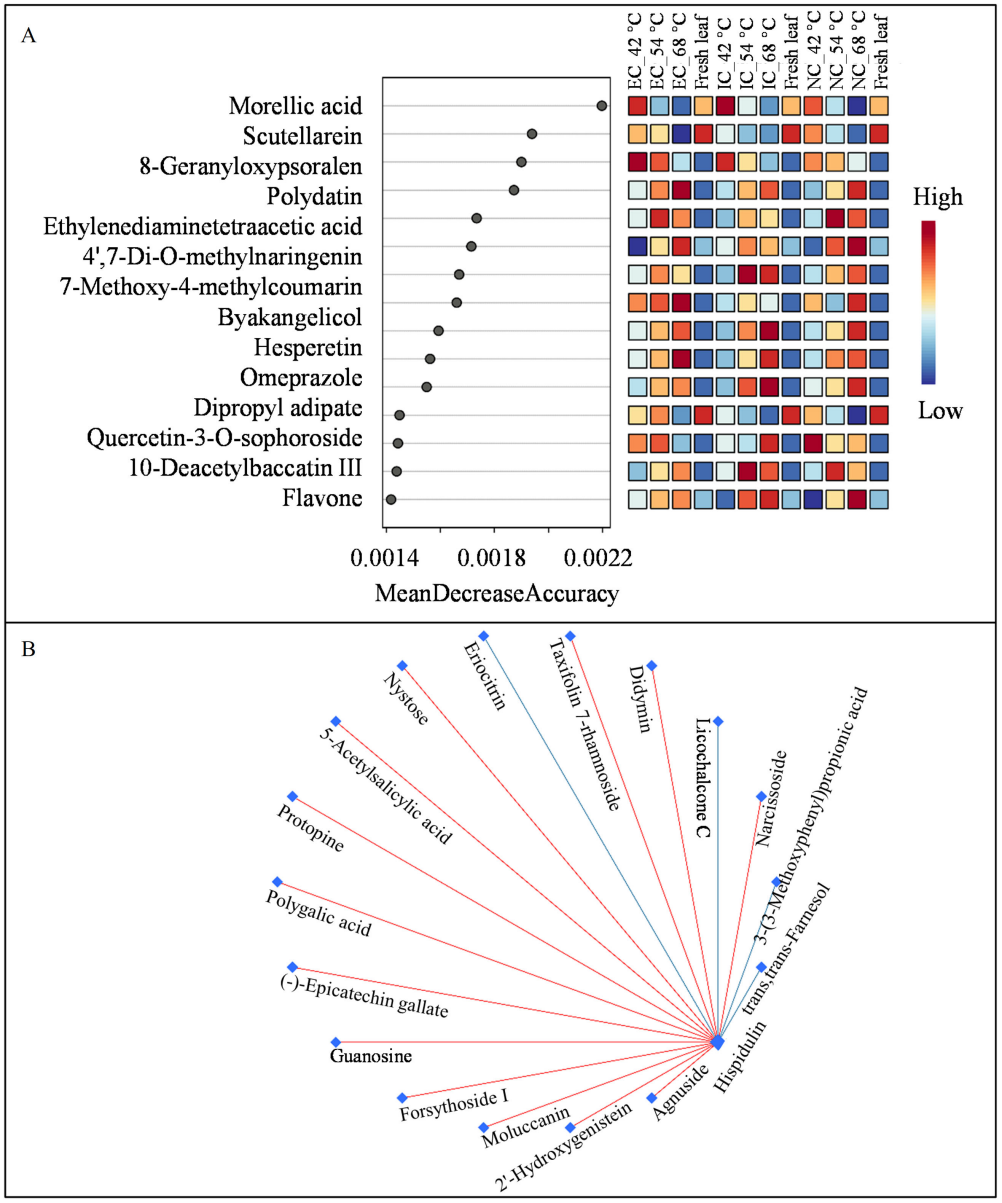
Analysis of metabolites by random forest **(A)** and debiased sparse partial correlation network **(B)**.

Random forest analysis, a powerful tool for identifying key variables in complex datasets, was employed to uncover the metabolic underpinnings of tobacco leaf responses to varying curing regimes (**Figure 6A**). Notably, morellic acid, a natural compound known for its antioxidant and anti-inflammatory properties (Aswathy et al., 2024), emerged as the core molecular metabolite across different treatment conditions and temperature variations. This finding underscores the potential of morellic acid as a biomarker for monitoring metabolic adjustments in tobacco leaves during curing.

### 2.7 Analysis of DSPC network

DPSC analysis provided a visualization of the correlation networks among metabolites. Flavonoids, known for their diverse biological functions, are the largest class of metabolites in tobacco leaves (Hu et al., 2021). Notably, hispidulin, an anticancer flavone, was identified as the core metabolite by DPSC among the seven isolated flavonoids (Rout et al., 2024). Hispidulin showed positive correlations with four flavonoids and negative correlations with two others (**Figure 6B**), indicating its potential value in elucidating the metabolic pathway of flavonoids.

## 3 Conclusions

Curing degrees (NC, EC, and IC) minimally affected the physicochemical properties of tobacco leaves, such as leaf density, leaf weight, and the content of nicotine, nitrogen, chlorine, and potassium. However, the sugar content varied among three curing degrees, notably showing that EC exhibited significantly less sugar compared to IC. LC-MS/MS identified 845 metabolites, with flavonoids predominant. Heatmap analysis indicated significant changes in metabolites throughout curing stages and treatments. Comparative analyses isolated 256 up-regulated and 241 down-regulated DEMs consistently changing from fresh leaf to cured leaves, with 91 DEMs demonstrating at least a 10-fold alteration, highlighting the substantial metabolite transformation during curing. Notably, nonivamide varied markedly across treatments, suggesting its potential as a key curing indicator. NC displayed 11 up- and 17 down-regulated unique DEMs compared to EC and IC, indicating their potential role in tobacco quality formation. KEGG pathway analysis indicated a significant shift in metabolic pathways during the critical stages from yellowing to color fixing, particularly those related to secondary metabolite biosynthesis and amino acid metabolism, potentially contributing to the desired flavor and aroma profiles. Correlation analysis isolated the top 25 DEMs correlated with curing degree and stage, which might play pivotal roles in the curing process and could serve as potential biomarkers for assessing curing degree and stage. Specifically, D-(+)-cellobiose displayed the strongest negative correlation with curing degree, followed by maltitol and androsin. Conversely, 5,7-dihydroxychromone exhibited the highest positive correlation coefficient, followed by ferulaldehyde and guanine. Furthermore, curcurbitacin IIa showed the highest positive correlation with curing stage, followed by hesperetin and 8-shogaol. Random forest analysis highlighted morellic acid as a consistent core metabolite across curing conditions, suggesting its potential as a biomarker. Moreover, DPSC analysis pinpointed hispidulin as a key metabolite, underlining its importance in flavonoid metabolism elucidation. Collectively, this study enhances the understanding of the metabolite transformation underlying tobacco curing processes and provides valuable insights for optimizing curing strategies to achieve desired product characteristics.

## 4 Materials and methods

### 4.1 Plant growth and sampling

Tobacco variety Yunyan 87 was cultivated at the research farm of Guizhou Academy of Tobacco Science in Fuquan City, Guizhou Province, China, situated at an altitude of 1200 meters belonging to subtropical monsoon climate. The soil composition consisted of 25.32 g/kg organic matter, 138.73 mg/kg of available nitrogen (N), 36.32 mg/kg of phosphorus (P), 218.69 mg/kg of potassium (K), and a pH value of 6.2. The experiment was conducted using a randomized block design, with each plot covering an area of 121 m^2^ and a planting density of 1.1 m × 0.55 m, replicated three times. Seeding was sown on January 20^th^, followed by transplantation on April 25^th^. The average temperatures ranged from 16.13°C to 23.17°C in May, 19.23°C to 24.93°C in June, 22.87°C to 29.83°C in July, and 21.30°C to 30.37°C in August. The base fertilizer regime included 525 kg/ha of compound fertilizer (with an N:P:K ratio of 10:10:25), 450 kg/ha of fermented oilseed meal and 375 kg/ha of calcium-magnesium-phosphate fertilizer. On the day of transplantation, each plant was treated with 150-200 mL of a water-soluble fertilizer solution, containing 1% compound fertilizer and 0.28% cyhalothrin emulsifiable concentrate. Ten days after transplantation, an additional 100-150 mL of water-soluble fertilizer, formulated with 4% compound fertilizer, was applied per plant. This application was repeated 30 days post-transplantation, maintaining the same volume and concentration of fertilizer. All cultivation and management measures were uniformly maintained in consistency.

The tobacco leaves from the middle section, which exhibited similar growth patterns and uniform leaf color and size, were prelabeled before sampling. At the maturation stage, 400 labeled leaves were sampled from each repetition and allocated to three different tobacco curing rooms to undergo normal curing (NC), excessive curing (EC), and insufficient curing (IC) process treatments, with the specific details outlined in **Table 4**.

**Table 4:** Parameters in the curing process of different treatments.

Treatment	Parameter	YS				CFS				DTS	
NC	Dry bulb (°C)	35	38	40	42	45	48	51	54	60	68
	Wet bulb (°C)	34	35	36	36	36	36	37	38	39	40
	Heating rate (°C/h)	1	1	0.5	0.5	0.5	0.5	0.5	0.5	1	1
	Time (h)	6	22	14	14	6	8	6	14	8	28
EC	Dry bulb (°C)	35	38	40	42	45	48	51	54	60	68
	Wet bulb (°C)	34	36	36	36	36	37	38	39	40	41
	Heating rate (°C/h)	1	1	0.5	0.5	0.5	0.5	0.5	0.5	1	1
	Time (h)	6	18	18	18	10	8	6	14	8	33
IC	Dry bulb (°C)	35	38	40	42	45	48	51	54	60	68
	Wet bulb (°C)	34	35	36	36	36	36	37	38	39	40
	Heating rate (°C/h)	1	1	0.5	0.5	0.5	0.5	0.5	0.5	1	1
	Time (h)	6	16	10	14	6	8	6	14	8	28

YS, yellowing stage; CFS, color fixing stage; DTS, dry tendon stage; NC, normal curing; EC, excessive curing; IC, insufficient curing.

The middle layers of fresh leaf and curing leaf at the end of yellowing (42°C), color fixing (54°C), and dry tendon (68°C) stages were collected in triplicate, with each repetition containing 30 pieces, respectively. Samples were stored at -80°C in an ultra-low temperature refrigerator for further measurement.

### 4.2 Determination of physicochemical composition

At the end of the flue-curing process, the leaf weight was measured, and the leaf density was subsequently calculated as the ratio of leaf weight to leaf area. The measurement of total sugar and chlorine contents was carried out in strict adherence to the “Tobacco and Tobacco Products - Determination of Water Soluble Sugars - Continuous Flow Method” (YC/T 159-2002) and the “Tobacco and Tobacco Products - Determination of Nicotine - Continuous Flow Method” (YC/T 160-2002), respectively. The determination of total nitrogen, potassium, and chlorine elements was conducted using the “Tobacco and Tobacco Products - Determination of Total Nitrogen - Continuous Flow Method” (YC/T 161-2002), “Tobacco and Tobacco Products - Determination of Potassium - Continuous Flow Method” (YC/T 217-2007), and the “Tobacco and Tobacco Products - Determination of Chlorine - Continuous Flow Method” (YC/T 162-2011), respectively.

### 4.3 LC-MS/MS analysis

A 150 mg sample was precisely weighed and ground in a 2 mL thick-walled tube using 1 mL of pre-cooled (-20°C) 7:3 methanol:water extraction solution. This mixture was stored at 4°C with periodic vortex mixing (every 10 min, 3 times) followed by overnight incubation to ensure thorough extraction. For LC-MS analysis, supernatants were filtered through 0.22 μm filter membrane and analyzed using a Waters ACQUITY UPLC I-Class Plus coupled with a QTRAP 6500 Plus mass spectrometer. Chromatographic separation was achieved on an HSS T3 column (2.1 mm x 10 cm, 1.8 μm, Waters) with a gradient mobile phase comprising 0.1% formic acid in water (A) and 0.1% formic acid in acetonitrile (B), selected to optimize separation efficiency and peak shape. The elution gradient was set as follows: 0 - 2.00 min, 5% B; 2.00 - 22.00 min, 5% B; 22.00 - 27.00 min, 95% B; 27.00 - 27.10 min, 95% B; 27.10 - 30.00 min, 5% B,at a flow rate of 0.300 ml/min with a column temperature maintained at 40°C (Bian et al., 2023).

For the QTRAP 6500 Plus equipped with ESI Turbo ion spray, ion source parameters were optimized as follows: ion source temperature, 450°C; ion spray voltage (IS), 5500 V (positive mode) and -4500 V (negative mode); ion source gas I (GS1), gas II (GS2), and curtain gas (CUR) set to 40, 40, and 20 psi, respectively. Multiple Reaction Monitoring (MRM) methods were configured in MRM mode, encompassing information on MRM transitions, collision energy (CE), DE clustering potential (DP), and retention time of target metabolites.

Metabolites were identified and quantified using Skyline software (version 21.1.0.146) in conjunction with the Beijing Genomics Institute (BGI)-Wide Target-Library database. Subsequent bio-informatics analysis encompassed comprehensive data preprocessing, data quality control, global analysis, and in-depth screening for inter-group differences across comparative groups using the online platforms MetaboAnalyst 6.0 (https://www.metaboanalyst.ca/) (Pang et al., 2024) and BGI (https://biosys.bgi.com/). Differential ex-pressed metabolites (DEMs) were identified by VIP ≥ 1, fold change values of ≥ 1.20 or ≤ 0.80, and p values ≤ 0.05. Hierarchical cluster analysis (HCA) was performed on samples and metabolites, with results presented as heatmaps accompanied by dendrograms. Pearson correlation coefficients (PCC) between samples were calculated using the cor function in R. Both HCA and PCC were performed using the pheatmap R package. Correlation Networks (DSPC) and Random Forest were generated by the online platform of MetaboAnalyst 6.0 (https://www.metaboanalyst.ca/) (Pang et al., 2024). Metabolites were annotated using the KEGG compound database (http://www.kegg.jp/kegg/compound/), and then mapped to the corresponding pathways in the KEGG pathway database (http://www.kegg.jp/kegg/pathway.html) (Kanehisa et al., 2016).

### 4.4 Data analysis

Data were presented as mean ± standard deviation based on three independent replicate experiments. Statistical significance at p ≤ 0.05 was determined using One-way ANOVA in SPSS version 18.0.

## Data Availability

The original contributions presented in the study are included in the article/**Supplementary Material**. Further inquiries can be directed to the corresponding author.

## References

[B1] Abubakar Y. Young J. Johnson W. Weeks W. (2000). Changes in moisture and chemical composition of flue-cured tobacco during curing. Tob. Sci. 44, 51–58. doi: 10.3381/0082-4623-44.1.51

[B2] Aswathy S. Joe I. H. Rameshkumar K. Ibrahim J. M. (2024). Insights into the role of RAHB and IHB interactions for the structure-activity relationship of caged xanthone morellic acid: Spectroscopic and DFT exploration, molecular docking, and molecular dynamics simulation studies as an Antituberculosis agent. J. Mol. Struct. 1303, 137429. doi: 10.1016/j.molstruc.2023.137429

[B3] Attoe O. (1946). Leaf-burn of tobacco as influenced by content of potassium, nitrogen, and chlorine. Agron. J. 38, 186–196. doi: 10.2134/agronj1946.00021962003800020009x

[B4] Bacon C. W. Wenger R. Bullock J. F. (1952). Chemical changes in tobacco during flue-curing. Indust. Eng. Chem. 44, 292–296. doi: 10.1021/ie50506a021

[B5] Banožić M. Jokić S. Ačkar Đ. Blažić M. Šubarić D. (2020). Carbohydrates-key players in tobacco aroma formation and quality determination. Molecules 25, 1734. doi: 10.3390/molecules25071734 32283792 PMC7181196

[B6] Bian J. Sun J. Chang H. Wei Y. Cong H. Yao M.. (2023). Profile and potential role of novel metabolite biomarkers, especially indoleacrylic acid, in pathogenesis of neuromyelitis optica spectrum disorders. Front. Pharmacol. 14. doi: 10.3389/fphar.2023.1166085 PMC10263123 37324490

[B7] Chen J. Li Y. He X. Jiao F. Xu M. Hu B.. (2021). Influences of different curing methods on chemical compositions in different types of tobaccos. Ind. Crops Prod. 167, 113534. doi: 10.1016/j.indcrop.2021.113534

[B8] Chen Y. Ren K. He X. Gong J. Hu X. Su J.. (2019). Dynamic changes in physiological and biochemical properties of flue-cured tobacco of different leaf ages during flue-curing and their effects on yield and quality. BMC Plant Biol. 19, 555. doi: 10.1186/s12870-019-2143-x 31842767 PMC6916008

[B9] Demole E. Enggist P. (1975). A chemical study of burley tobacco flavour (*Nicotiana tabacum* L.) VI. Identification and synthesis of four irregular terpenoids related to solanone, including a ‘Prenylsolanone’. Helv. Chim. Acta 58, 1602–1607. doi: 10.1002/hlca.19750580614 1176296

[B10] Gao L. Gao J. M. Ren X. H. Gao Y. B. Huang G. H. Wang X.. (2023). Effects of poly-γ-glutamic acid (γ-PGA) on metabolites of flue-cured tobacco leaves based on metabolomics analysis. Russ. J. Plant Physiol. 70, 140. doi: 10.1134/S1021443723601647

[B11] Geng Z. Yang H. Gao H. Xing L. Hu X. Yan T.. (2024). Metabolomics reveal the chemical characteristic of cigar tobacco leaves during air-curing process. J. Biobased. Mater. Bioenergy 18, 621–633. doi: 10.1166/jbmb.2024.2411

[B12] Gong Y. Li J. Deng X. Chen Y. Chen S. Huang H.. (2023). Application of starch degrading bacteria from tobacco leaves in improving the flavor of flue-cured tobacco. Front. Microbiol. 14. doi: 10.3389/fmicb.2023.1211936 PMC10335769 37440887

[B13] Henry J. B. Vann M. C. Lewis R. S. (2019). Agronomic practices affecting nicotine concentration in flue-cured tobacco: A review. Agron. J. 111, 3067–3075. doi: 10.2134/agronj2019.04.0268

[B14] Higashio Y. Shoji T. (2004). Heterocyclic compounds such as pyrrole, pyridines, pyrrolidine, piperidine, indole, imidazol and pyrazines. Appl. Catal. A Gen. 260, 251–259. doi: 10.1016/S0926-860X(03)00197-2

[B15] Hu Z. Pan Z. Yang L. Wang K. Yang P. Xu Z.. (2021). Metabolomics analysis provides new insights into the medicinal value of flavonoids in tobacco leaves. Mol. Omics 17, 620–629. doi: 10.1039/D1MO00092F 34137416

[B16] Kanehisa M. Sato Y. Kawashima M. Furumichi M. Tanabe M. (2016). KEGG as a reference resource for gene and protein annotation. Nucleic Acids Res. 44, 457–462. doi: 10.1093/nar/gkv1070 PMC4702792 26476454

[B17] Li J. Ma Z. Dai H. Li H. Qiu J. Pang X. (2024). Application of PLSR in correlating sensory and chemical properties of middle flue-cured tobacco leaves with honey-sweet and burnt flavour. Heliyon 10, e29547. doi: 10.1016/j.heliyon.2024.e29547 38655300 PMC11035049

[B18] Li N. Yu J. Yang J. Wang S. Yu L. Xu F.. (2023). Metabolomic analysis reveals key metabolites alleviating green spots under exogenous sucrose spraying in air-curing cigar tobacco leaves. Sci. Rep. 13, 1311. doi: 10.1038/s41598-023-27968-8 36693869 PMC9873923

[B19] Li X. Bin J. Yan X. Ding M. Yang M. (2022). Application of chromatographic technology to determine aromatic substances in tobacco during natural fermentation: A review. Separations 9, 187. doi: 10.3390/separations9080187

[B20] Li Y. Wang Y. Ma W. Tan C. (2001). Breeding and selecting of a new flue-cured tobacco variety Yunyan87 and its characteristics. Chin. Tobacco. Sci. 22, 38–42. doi: 10.3969/j.issn.1007-5119.2001.04.003

[B21] Lim H. H. Choi K. Y. Shin H. S. (2022). Flavor components in tobacco capsules identified through non-targeted quantitative analysis. J. Mass. Spectrom. 57, e4811. doi: 10.1002/jms.4811 35088484

[B22] Liu J. Wang J. Du Y. Yan N. Han X. Zhang J.. (2024). Application and evaluation of the antifungal activities of glandular trichome secretions from air/sun-cured tobacco germplasms against *botrytis cinerea* . Plants 13, 1997. doi: 10.3390/plants13141997 39065524 PMC11280957

[B23] Mavroeidis A. Stavropoulos P. Papadopoulos G. Tsela A. Roussis I. Kakabouki I. (2024). Alternative crops for the European tobacco industry: A systematic review. Plants 13, 236. doi: 10.3390/plants13020236 38256796 PMC10818552

[B24] Meng L. Song W. Chen S. Hu F. Pang B. Cheng J.. (2022). Widely targeted metabolomics analysis reveals the mechanism of quality improvement of flue-cured tobacco. Front. Plant Sci. 13. doi: 10.3389/fpls.2022.1074029 PMC9746875 36523627

[B25] Meng Y. Wang Y. Guo W. Lei K. Chen Z. Xu H.. (2024). Analysis of the relationship between color and natural pigments of tobacco leaves during curing. Sci. Rep. 14, 166. doi: 10.1038/s41598-023-50801-1 38167588 PMC10762081

[B26] Pang Z. Lu Y. Zhou G. Hui F. Xu L. Viau C.. (2024). MetaboAnalyst 6.0: Towards a unified platform for metabolomics data processing, analysis and interpretation. Nucleic Acids Res. 52, gkae253. doi: 10.1093/nar/gkae253 PMC11223798 38587201

[B27] Rout K. K. Kar M. K. Agarwal P. C. Dash S. K. (2024). Analysis of bioactive hispidulin: an anticancer flavone of *Clerodendrum philippinum.* J. Planar. Chromat. 37, 49–56. doi: 10.1007/s00764-023-00267-8

[B28] Shoji T. Hashimoto T. Saito K. (2024). Genetic regulation and manipulation of nicotine biosynthesis in tobacco: strategies to eliminate addictive alkaloids. J. Exp. Bot. 75, 1741–1753. doi: 10.1093/jxb/erad341 37647764 PMC10938045

[B29] Sun C. L. Zhang H. L. Zhou D. B. Cheng Z. J. Xie Y. Rang Z. W.. (2023). Based on metabolomics, the optimum wind speed process parameters of flue-cured tobacco in heat pump bulk curing barn were explored. Sci. Rep. 13, 21558. doi: 10.1038/s41598-023-49020-5 38057437 PMC10700593

[B30] Vaughan C. Stanfill S. B. Polzin G. M. Ashley D. L. Watson C. H. (2008). Automated determination of seven phenolic compounds in mainstream tobacco smoke. Nicotine. Tob. Res. 10, 1261–1268. doi: 10.1080/14622200802123146 18629737

[B31] Wang J. Liu S. M. Long J. Lei D. A. Gao F. (2020). Derivatization method for the determination of amino acids in tobacco by gas chromatography-mass spectrometry. J. Anal. Chem. 75, 1046–1053. doi: 10.1134/S1061934820080171

[B32] Weng S. Deng M. Chen S. Yang R. Li J. Zhao X.. (2024). Application of pectin hydrolyzing bacteria in tobacco to improve flue-cured tobacco quality. Front. Bioeng. Biotechnol. 12. doi: 10.3389/fbioe.2024.1340160 PMC10955059 38515623

[B33] Xin X. Gong H. Hu R. Ding X. Pang S. Che Y. (2023). Intelligent large-scale flue-cured tobacco grading based on deep densely convolutional network. Sci. Rep. 13, 11119. doi: 10.1038/s41598-023-38334-z 37429961 PMC10333347

[B34] Yang H. Sun G. Yin G. Sun H. Wang T. Bai T.. (2023). Browning mechanism of tobacco leaves during flue-curing process: Proteomics and metabolomics analysis reveals the changes in materials. Mater. Express. 13, 1068–1080. doi: 10.1166/mex.2023.2443

[B35] Zhao Y. Zhao J. Zhao C. Zhou H. Li Y. Zhang J.. (2015). A metabolomics study delineating geographical location-associated primary metabolic changes in the leaves of growing tobacco plants by GC-MS and CE-MS. Sci. Rep. 5, 16346. doi: 10.1038/srep16346 26549189 PMC4637841

[B36] Zong J. He X. Lin Z. Hu M. Xu A. Chen Y.. (2022). Effect of two drying methods on chemical transformations in flue-cured tobacco. Drying. Technol. 40, 188–196. doi: 10.1080/07373937.2020.1779287

[B37] Zou X. Bk A. Rauf A. Saeed M. Al-Awthan Y. S. Al-Duais M. A.. (2021). Screening of polyphenols in tobacco (*Nicotiana tabacum*) and determination of their antioxidant activity in different tobacco varieties. ACS Omega. 6, 25361–25371. doi: 10.1021/acsomega.1c03275 34632194 PMC8495694

[B38] Zou C. Hu X. Huang W. Zhao G. Yang X. Jin Y.. (2019). Different yellowing degrees and the industrial utilization of flue-cured tobacco leaves. Sci. Agric. 76, 1–9. doi: 10.1590/1678-992x-2017-0157

[B39] Zou L. Su J. Xu T. Ji X. Wang T. Chen Y.. (2023). Untargeted metabolomics revealing changes in aroma substances in flue-cured tobacco. Open Chem. 21, 20220326. doi: 10.1515/chem-2022-0326

